# Low-intensity pulsed ultrasound in obstetrics and gynecology: advances in clinical application and research progress

**DOI:** 10.3389/fendo.2023.1233187

**Published:** 2023-07-31

**Authors:** Xiaoyu Ji, Hua Duan, Sha Wang, Yanan Chang

**Affiliations:** Department of Minimally Invasive Gynecology, Beijing Obstetrics and Gynecology Hospital, Capital Medical University, Beijing Maternal and Child Health Care Hospital, Beijing, China

**Keywords:** LIPUS, tissue repair, biological mechanisms, clinical application, obstetrics and gynecology

## Abstract

In the past decade, research on ultrasound therapy in obstetrics and gynecology has rapidly developed. Currently, high-intensity ultrasound has been widely used in clinical practice, while low-intensity ultrasound has gradually emerged as a new trend of transitioning from pre-clinical research to clinical applications. Low-intensity pulsed ultrasound (LIPUS), characterized by a non-invasive low-intensity pulse wave stimulation method, employs its non-thermal effects to achieve safe, economical, and convenient therapeutic outcomes. LIPUS converts into biochemical signals within cells through pathways such as cavitation, acoustic flow, and mechanical stimulation, regulating molecular biological mechanisms and exerting various biological effects. The molecular biology mechanisms underlying the application of LIPUS in obstetrics and gynecology mainly include signaling pathways, key gene expression, angiogenesis, inflammation inhibition, and stem cell differentiation. LIPUS plays a positive role in promoting soft tissue regeneration, bone regeneration, nerve regulation, and changes in cell membrane permeability. LIPUS can improve the treatment benefit of premature ovarian failure, pelvic floor dysfunction, nerve damage caused by intrauterine growth restriction, ovariectomized osteoporosis, and incomplete uterine involution through the above biological effects, and it also has application value in the adjuvant treatment of malignant tumors such as ovarian cancer and cervical cancer. This study outlines the biological mechanisms and applications of LIPUS in treating various obstetric and gynecologic diseases, aiming to promote its precise application and provide a theoretical basis for its use in the field.

## 1 Introduction

Over recent decades, ultrasound has evolved from a diagnostic imaging modality to therapeutic applications, encompassing both high-intensity and low-intensity ultrasound (1). High-intensity focused ultrasound (HIFU) has gained widespread use in obstetrics and gynecology for treating various benign and malignant tumors, particularly uterine fibroids and adenomyosis. HIFU induces coagulation necrosis of target tissues without damaging the surrounding normal tissues in producing instantaneous high thermal effects and mechanical effects (2). In contrast, low-intensity pulsed ultrasound (LIPUS) affects cellular material metabolism processes, accelerates tissue metabolism, improves ischemia and hypoxia states, enhances tissue nutrition, and promotes tissue repair through minimal thermal effects and significant non-thermal effects in the target tissues (3). The non-thermal effects of LIPUS predominantly involve cavitation-induced micro-bubbles and microjets, acoustic flow, and mechanical stimulation that convert into biochemical signals within cells to produce biological effects (4). Tissue absorption of ultrasonic energy is essential for exerting biological effects, with different tissues exhibiting diverse absorption capacities for ultrasonic waves. Highly protein-rich and low-water content tissues absorb ultrasonic energy to a greater extent, with bone and cartilage exhibiting the highest energy absorption, followed by tendon, skin, muscle, nerve, fat, and blood (5).

**Figure 1:**
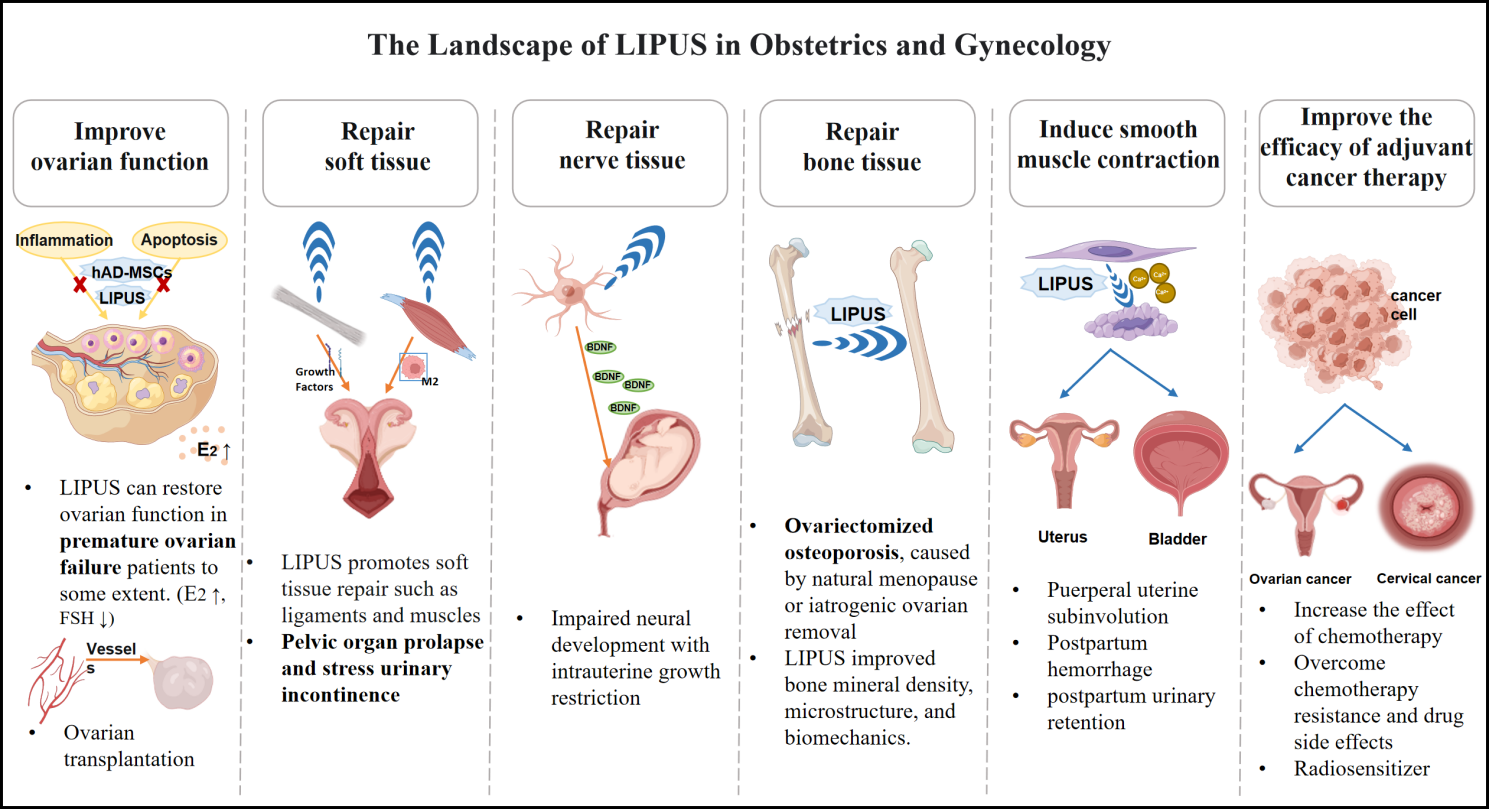
The overview of LIPUS playing a therapeutic role in the field of obstetrics and gynecology.

LIPUS is a type of medium-frequency ultrasound (0.7-3 MHz) that is pulsed in wave mode (100 and 1,000 Hz) and delivered at an intensity (<3 W/cm2) much lower than traditional ultrasound energy (6). Most piezoelectric transducers on the market are made of ceramic materials that can convert input electrical energy into mechanical energy (ultrasound waves). When the ultrasound beam is emitted from the therapeutic ultrasound device’s treatment head, the energy distribution in space within the beam is non-uniform (7). The ultrasound beam closest to the treatment head is called the near field, where the ultrasound energy is higher and varies significantly locally, making it more commonly used in LIPUS therapy applications. The length of the near field is influenced by the transducer radius, speed of sound in the medium, and frequency, which allows for changes in the size and shape of ultrasound transducers to meet the treatment needs of different parts (8). LIPUS is a safe, economical and convenient treatment modality that has been demonstrated to effectively promote healing of surgical incisions, fractures, tendon injuries, and nerve damage (4). The above applications reflect the LIPUS repair of nerves, blood vessels, muscles and other soft tissues and the control of inflammation, which is closely related to many gynecological diseases such as ovarian function decline, pelvic floor dysfunction, and incomplete uterine involution after delivery. In recent years, with the deepening of research on the effects of ultrasound in biology, LIPUS has gradually received widespread attention in the field of obstetrics and gynecology, indicating a broad application prospect (**Figure 1**). Currently, there is no systematic review summarizing the research progress of LIPUS in the field of obstetrics and gynecology. Thus, this study systematically summarizes the application direction and research advancements of LIPUS in obstetrics and gynecology to promote further LIPUS-related research in these fields.

## 2 LIPUS improves ovarian function

As a kind of mechanical sound wave, ultrasound penetrates the tissue directly to reach the treatment site, which can be used to improve the pelvic microcirculation, promote tissue nutrition, and accelerate the repair and regeneration of ovarian tissue. As early as in the 1970s, Terhaar et al. (9) found that a small dose of ultrasound could promote the formation of ovarian follicles in mice. However, this finding was not further studied. Until the rapid development of ultrasound therapy in recent years, the impact of ultrasound therapy on ovarian function has come back to the research field.

### 2.1 LIPUS improves the condition of premature ovarian failure

Premature ovarian failure (POF) is the onset of ovarian failure due to ovarian follicle depletion or iatrogenic injury before the age of 40 years. The main causes include genetic factors, autoimmune diseases and iatrogenic injury, etc., and the pathological characteristics are hypomenorrhea or amenorrhea, increased gonadotropin levels (FSH > 25 IU/L) and decreased estradiol (10). In 2021, a team reported that LIPUS was beneficial to the treatment of cyclophosphamide induced ovarian injury in rats. The results showed that the ultrasound treatment group had fewer atretic follicles, reduced gonadotropin levels, and increased estrogen levels, suggesting that LIPUS could restore ovarian function in POF patients to some extent (11). In the above study, LIPUS had no significant effect on serum anti-Mullerian hormone (AMH) levels and pregnancy rate, but in another model of 4- vinylcyclohexene diepoxide (VCD) -induced ovarian injury in rats, AMH levels were significantly increased after LIPUS treatment (12), this result may be affected by factors such as observation time and sample size. Notably, inflammation and apoptosis play an important role in normal reproduction, and inhibition of inflammatory and apoptotic pathways can alleviate ovarian damage (13). Interestingly, LIPUS reversed the apoptosis and inflammatory state of VCD-induced ovarian injury, resulting in corresponding changes at the cellular structural level, such as the reduction of ovarian cell structural damage, autophagosome occurrence, and endoplasmic reticulum expansion (12).

In addition, mesenchymal stem cell (MSC) transplantation is an effective treatment for POF and has been shown to restore ovarian structure and function in animal models (14). Human amnion-derived mesenchymal stem cells (hAD-MSCs) have been shown to possess the characteristics of MSC (15). The paracrine proteome of hAD-MSCs in the ovarian microenvironment can protect the ovary from iatrogenic injury by reducing apoptosis and promoting angiogenesis, cell proliferation and gene expression (16). LIPUS has been verified to promote the survival and proliferation of hAD-MSCs, and increase the ratio of the anti-apoptotic protein Bcl-2 to pro-apoptotic protein Bax, showing the benefit of the anti-apoptotic effect (17, 18). LIPUS combined with hAD-MSCs has been studied in the treatment of POF. Compared with hAD-MSC transplantation alone, the secretion of stem cell-related factors in the LIPUS pretreatment group increased, which was more conducive to reducing the inflammatory response of ovarian tissue in rats with POF and improving the local microenvironment (18). At the same time, LIPUS can promote the expression of chemokine receptor CXCR 4 in hAD-MSC, further increasing the number of migration and homing of hAD-MSC to the ovary (19). These findings provide evidence that LIPUS combined with stem cell therapy is effective in improving ovarian function in patients with POF.

### 2.2 LIPUS benefits the outcome of ovarian transplantation

One of the important challenges faced by ovarian transplantation is ischemia-reperfusion injury, which may destroy 30% to 70% of primordial follicles (20). Therefore, the rapid establishment of vascular connections is one of the important factors for successful transplantation and is crucial for follicle survival (21). LIPUS can accelerate angiogenesis and promote tissue repair and enhance regeneration. Previous studies have demonstrated that LIPUS treatment can promote angiogenesis, significantly improve capillary density and myocardial blood flow at the ischemic site, and reduce the degree of myocardial fibrosis in the porcine ischemic cardiomyopathy model (22, 23). In a study of capsule tissues in rabbits, tissue perfusion, hemoglobin content, and vascular density were significantly higher in the LIPUS treatment group, which promoted angiogenesis by stimulating the release of VEGF-α and bFGF, two essential growth factors involved in angiogenesis, from endothelial cells (24). In addition, LIPUS may lead to enhanced angiogenesis by increasing the expression of endothelial nitric oxide synthase, phosphorylated ERK, phosphorylated Akt and phosphorylated YAP protein (23, 25).

From Iran’s biomedical research team, through the animal experiment research LIPUS on ectopic mice ovarian follicle development, angiogenesis and apoptosis after transplantation. The results showed that there was a higher expression of CD31 vascular endothelial growth factor in the LIPUS group, indicating a greater number of blood vessels generated. Ultrasound therapy could effectively accelerate blood vessel formation and reduce ischemic injury (especially within the first 48 hours after transplantation), thus better maintaining the function of transplanted ovaries and reducing ovarian cell apoptosis (20). Therapeutic LIPUS can accelerate and increase angiogenesis, improve inflammation and enhance healing after ovarian tissue transplant stress, and help promote follicular growth in the transplanted ovary. The benefits of LIPUS on ovarian function have been preliminaries confirmed, but most of the current research is still in the stage of animal experiments, so further clinical trails need to make its real applicatiaon in clinical practice.

## 3 LIPUS promotes the repair of multiple soft tissues

Soft tissue refers to non-mineralized tissues or organs in living organisms and can be divided into two main categories: connective tissue and non-connective tissue. Tendons, skin, fat, and ligaments belong to connective tissues, while blood vessels, nerves, and muscles belong to non-connective tissues (26). Micro-energy therapy can regulate the polarization of macrophages, reduce inflammation and promote tissue repair by reducing the secretion of inflammatory factors such as IL-1β, IL-6, and IL-8 (27, 28). It can also play a key role in the repair of a variety of diseases caused by soft tissue injury by regulating the expression of growth factors VEGF-α, bFGF and TGF-β (24, 29).

Pelvic organ prolapse (POP) is the displacement of pelvic organs caused by weakened pelvic support, which includes muscles, fascia, and ligaments. The main causes of POP are injury due to pregnancy and childbirth, as well as structural atrophy resulting from decreased estrogen levels after menopause. LIPUS treatment is beneficial to the recovery of damaged muscle function, which has been indirectly proved by the results of multiple comprehensive functional tests, and its therapeutic effect has also been directly confirmed by muscle mechanical tests. Both clinical trials and preclinical studies have shown that LIPUS can reduce inflammatory response and accelerate muscle injury recovery, and this beneficial change may be related to increased blood flow, activated mitochondrial biogenesis, and anti-oxidative stress effects (30). Qin et al. (30) found that LIPUS with an intensity of 60 mW/cm2 can promote muscle regeneration significantly by exerting an anti-inflammatory effect through inducing macrophage phenotypic switching from M1 to M2, possibly achieved by upregulating FZD5 expression in the WNT pathway and enhancing nuclear translocation of β-catenin in macrophages. In addition, muscle atrophy is often accompanied by muscle tissue protein metabolism disorders, increased muscle protein breakdown, muscle fiber thinning or even disappearance, and ultrasound therapy can promote protein synthesis in the tissue (31), the key mechanism may be related to promoting protein synthesis and stabilizing alanine, aspartic acid and glutamate metabolism through the MSTN/Akt/mTOR signaling pathway (32). Animal models have also demonstrated that LIPUS treatment can accelerate ligament repair by significantly increasing tissue collagen content (33). Meanwhile, POP not only has the change of pelvic floor support structure, but also includes the damage of pelvic floor nerves and blood vessels. LIPUS stimulation can repair a variety of soft tissue injuries, suggesting that it has a certain therapeutic effect on POP.

Stress urinary incontinence (SUI) is a common concomitant symptom of uterine prolapse, which is one of the manifestations of female pelvic floor dysfunction. At present, there is a lack of effective evidence to confirm the long-term efficacy of drug treatment, and surgery cannot restore the underlying pathological changes of the disease. To demonstrate that ultrasound therapy is a potential treatment for SUI, Yang et al. (34) established a rat model of SUI and treated it with different LIPUS parameters in 2019. The results showed that LIPUS with appropriate parameters could improve the clinical symptoms of SUI. Compared with the SUI non-treatment group, the leakage point pressure and bladder capacity of the 200 mW/cm2 and 300 mW/cm2 groups were restored, and the proliferation and myoblast differentiation of rat skeletal muscle satellite cells were obvious. LIPUS-stimulated regeneration can increase the thickness and integrity of the urethral striated muscle and may play a positive role in relieving the symptoms of SUI.

## 4 LIPUS promotes the repair of nerve damage

LIPUS is a non-interventional stimulation method characterized by low-intensity pulse waves, which can promote the regeneration of nerve damage. LIPUS can repair peripheral nerve damage and exert neuroprotective effects by affecting nerve tissue engineering seed cells, neurotrophic factors, and nerve conduits (35, 36). In the brain, LIPUS increases the electrical activity of cortical neurons without causing any damage to the brain tissue. Previous studies have demonstrated neuroprotective effects of LIPUS in animal models of traumatic brain injury (37), cerebral ischemia (38), and vascular dementia (39). The mechanisms of neuroprotective effects of LIPUS are multimodal and include enhanced neurotrophic factor release, anti-inflammation, and anti-apoptosis (40).

Survivors of intrauterine growth restriction (IUGR) are at increased risk of impaired neurodevelopment, which may lead to lifelong motor and cognitive impairment, resulting in neurological deficits that currently have no treatment to cure (41, 42). Interventions that increase brain growth factors such as BDNF have therapeutic effects on IUGR-induced brain injury. Prenatal LIPUS treatment may reduce IUGR-induced brain damage by enhancing BDNF/CaMKII/Akt signaling in the offspring brain and increasing BDNF and GLUT 1 expression in the placenta (43). At the same time, maternal LIPUS treatment in IUGR rat model can also increase maternal weight, fetal weight and placental weight, and improve the placental function and fetal growth of IUGR rat model. These data provide a new evidence that LIPUS stimulation can be used as a neuroprotective treatment for IUGR and also has bene fits for IUGR-induced malnrtrition, which is a new technology worthy of attention in perinatal medicine.

## 5 LIPUS promotes the repair of bone tissue

LIPUS is effective in promoting fracture healing and treating osteoporosis, although its mechanism remains unclear. Studies have shown that LIPUS can significantly improve the mechanical microenvironment of bone trabeculae and osteoblasts through mechanical stimulation, promoting bone tissue repair (44). The combination of LIPUS with cell therapy based on hydrogels has a better promoting effect on osteoblasts and has been approved by the FDA as a method for fracture repair (45). Therefore, it is reasonable to speculate that LIPUS may have therapeutic potential for osteoporosis caused by natural menopause or ovarian function loss due to medical reasons. LIPUS with an intensity of 30 mW/cm2 was sufficient to promote bone defect healing in an ovariectomized rat model of osteoporosis. After 6 weeks of intervention, 150 mW/cm2 LIPUS showed improvement in bone mineral density, microstructure, and biomechanics compared with 30 mW/cm2, indicating that higher intensity LIPUS is more beneficial for osteoporotic bone repair (46). MSTN is a TGF-β family member that acts as a negative regulator of skeletal muscle growth. MSTN deficiency also has a positive effect on bone formation. Experiments have confirmed that LIPUS can effectively reduce the MSTN content in serum and quadriceps muscle of ovariectomized rats, which suggests that LIPUS may improve osteoporosis and promote bone defect healing in ovariectomized rats by inhibiting the MSTN signaling pathway (47). These evidences suggest that LIPUS can benefit women with ovariectomized osteoporosis, but further safe and reliable clinical trials need to be designed.

## 6 LIPUS induces smooth muscle contraction

Ultrasonic has mechanical effect, thermal effect and physical and chemical effect, biological effect is complex. LIPUS activates calcium (Ca^2+^) -dependent transcription factors cAMP-responsive element-binding protein and nuclear factor of activated T cells, which significantly increasing Ca^2+^ influx and increasing intracellular Ca^2+^ levels (48), which provides a theoretical basis for LIPUS-induced smooth muscle contraction.

Terhaar et al. (9) first found that ultrasound (3 MHz,2 W/cm2) can cause uterine contractions in pregnant mice, and can increase the frequency and amplitude of spontaneous contractions of the uterus. LIPUS induction of uterine smooth muscle contraction may be utilized to promote uterine involution and prevent postpartum hemorrhage. The results of a multicenter, randomized, controlled clinical trial showed that the postpartum women in the LIPUS group were superior to the control group in reducing fundus uteri height, shortening lochia duration, and relieving postpartum pain, with significant differences between the two groups, and no treatment-related adverse reactions and adverse events were observed (49). Therefore, LIPUS is a safe and effective method for treating postpartum uterine involution. Possible reasons why LIPUS can promote postpartum uterine involution include: (1) The mechanical effect of ultrasound can produce mechanical stimulation, and the uterine smooth muscle tissue is very sensitive to a certain frequency of mechanical stimulation and can produce contraction; (2) LIPUS slightly increases the tissue temperature, which may affect the metabolic activity of cells by increasing the enzyme activity; (3) LIPUS changes the permeability of the cell membrane to Ca^2+^, thereby promoting the increase of intracellular Ca^2+^ level and initiating the excitation-contraction coupling process of smooth muscle.

LIPUS can also accelerate bladder smooth muscle contraction by opening L-type calcium channels and activating Ca^2+^ signaling pathways. A retrospective study analyzed the records of 136 patients with postpartum urinary retention (PUR) in two different treatment groups receiving LIPUS and neostigmine, and the results showed that LIPUS had a higher response rate to PUR (80.6% vs. 64.1%, p<0.05), and no early or late adverse events were observed. In addition, The use of nimodipine (Ca^2+^ inhibitor) demonstrated that ultrasound promoted bladder smooth muscle contraction through activation of Ca^2+^ signaling pathways and increased troponin expression (50). Therefore, LIPUS is a safe and effective method for the treatment of PUR by inducing the contraction of bladder smooth muscle.

## 7 LIPUS improves the efficacy of cancer chemotherapy and radiotherapy

Ultrasound is a kind of non-ionizing mechanical wave, which has fewer adverse effects than traditional medical or surgical treatment. On the one hand, the cavitation effect of LIPUS within the tumor causes the rapid generation and rupture of micro-bubbles, generating mechanical shock waves, free radicals, and apoptotic initiators that directly inhibit cancer cell growth (51). The exact reason why LIPUS suppresses cancer cell proliferation may be due to pathways such as promoting cell apoptosis, necrosis, lysis, or disrupting the cell cycle (52, 53). On the other hand, current research focuses more on the combined use of LIPUS and other adjuvant therapies, which can play an important role in cancer treatment by enhancing the ability of anti-tumor drugs. Compared with normal cells, malignant cells are more sensitive to ultrasound irradiation due to their unique cell membrane properties. Therefore, ultrasound can selectively modify the membrane of diseased cells. The microjets produced by cavitation make the cancer cell membrane unstable, thereby increasing the uptake of drugs by cells, enhancing the effect of chemotherapy and targeted therapy, and minimizing the toxicity of drugs to nearby healthy cells (54). LIPUS plays an increasingly clear role in the treatment of cancer, and has also attracted great attention in gynecological cancer. Existing studies mainly focus on ovarian cancer and cervical cancer.

Currently, the main adjuvant treatment for ovarian cancer is chemotherapy, using paclitaxel combined with platinum drugs is the first-line regimen. Traditional cancer chemotherapy has limitations such as drug resistance and drug side effects. The effectiveness of LIPUS combined with platinum to overcome chemoresistance has been demonstrated in platinum-resistant ovarian cancer cells by measuring cell viability, colony formation, and cell cycle analysis (55). The principle may be that LIPUS improves cell membrane permeability and intracellular drug concentration increases the therapeutic effect. Recent studies in a 3D model of cisplatin resistance in ovarian cancer have shown that nanoparticles can further increase the therapeutic effect of ultrasound against resistance (56). The mechanism of action of paclitaxel is mainly to target microtubules, so as to inhibit cell mitosis and play an anti-cancer role. Amaya et al. (57) found that ultrasonic shock wave treatment temporarily disrupted the microtubule cytoskeleton and abolished taxol-induced rigid microtubule bundles. Based on the fact that transient exposure to LIPUS can reduce and/or eliminate the cytotoxicity associated with paclitaxel treatment of ovarian cancer cells, a strategy can be developed to combat the side effects of taxol-based chemotherapy in cancer patients. Local use of LIPUS only at the desired site to eliminate cytotoxicity without affecting the effect of paclitaxel on cancer cell activity is expected to be used to prevent chemotreatment-induced alopecism and peripheral neuropathy and improve the quality of life of patients (58). In addition, the above purpose can also be achieved with the help of molecular materials. Phase-changeable, folate-targeted perfluoropentane nanodroplets loaded with 10- hydroxycamptothecin and superparamagnetic Fe_3_O_4_ have been fabricated for multi-modality cancer imaging and targeted therapy. LIPUS-activated nanodroplets can improve the therapeutic effect on cancer cells and relatively reduce the side effects on normal tissues (59).

LIPUS can potentially play a role in radiotherapy and chemotherapy of cervical cancer. Transient mechanical effects mediated by acoustic pores generated by LIPUS enhance membrane permeability and disband the cytoskeleton early after acoustic pores. Cervical cancer HeLa cells were arrested in different cycle stages, and changes in membrane permeability and cytoskeletal arrangement induced by acoustic pore technology were simultaneously analyzed using real-time fluorescence imaging systems. The research showed that S-phase may be considered as the optimal cell cycle for transient acoustic perforation to promote gene/drug delivery therapy (60). The combination of S-phase blocking drugs with LIPUS may increase the efficacy of chemotherapy in treating cervical cancer. In addition, LIPUS can also be used as a sensitizer for radiotherapy. In HeLa cell experiments, radiation at a dose of 2 Gy combined with ultrasound treatment at any intensity (0.5, 1.0, 1.5 W/cm2) resulted in a significant decrease in cell survival compared to incubation for 72 h after radiation alone (61). Therefore, it is reasonable to believe that the combination therapy of LIPUS can enhance the effect of radiotherapy for cervical cancer. However, it needs further verification in subsequent animal experiments and clinical trials.

## 8 Summary and prospect

LIPUS is a promising non-invasive method that can promote various tissue repair, inhibit inflammatory response, and change cell membrane permeability through mechanical, thermal and physical and chemical effects. LIPUS has valuable applications in reproductive medicine, perinatal medicine, postpartum recovery, gynecological cancer and other directions. Although there is still insufficient understanding of the biological and biodynamic effects of LIPUS in human tissues and their effects on organs and the whole body, the therapeutic results obtained so far in the preclinical stage are very promising. These results may trigger a new research boom in the clinical field in the next few years. The future goals include studying the biological effects and molecular mechanisms of LIPUS on tissues and cells, screening treatment parameters suitable for different tissues, organs, and diseases, clarifying the treatment standards for various diseases, and effectively translating animal and cellular level studies into clinical applications.

## Author contributions

XJ conceived the study and edited the manuscript. HD, SW and YC edited and reviewed the manuscript. All authors contributed to the article and approved the submitted version.
